# Proteolysis-targeting chimera (PROTAC) for targeted protein degradation and cancer therapy

**DOI:** 10.1186/s13045-020-00885-3

**Published:** 2020-05-13

**Authors:** Xin Li, Yongcheng Song

**Affiliations:** 1 Department of Pharmacology and Chemical Biology, Baylor College of Medicine, 1 Baylor Plaza, Houston, TX 77030 USA; 2 Dan L. Duncan Comprehensive Cancer Center, Baylor College of Medicine, 1 Baylor Plaza, Houston, TX 77030 USA

**Keywords:** PROTAC, Targeted protein degradation, Cancer therapy, Hematological malignancies

## Abstract

Proteolysis-targeting chimera (PROTAC) has been developed to be a useful technology for targeted protein degradation. A bifunctional PROTAC molecule consists of a ligand (mostly small-molecule inhibitor) of the protein of interest (POI) and a covalently linked ligand of an E3 ubiquitin ligase (E3). Upon binding to the POI, the PROTAC can recruit E3 for POI ubiquitination, which is subjected to proteasome-mediated degradation. PROTAC complements nucleic acid-based gene knockdown/out technologies for targeted protein reduction and could mimic pharmacological protein inhibition. To date, PROTACs targeting ~ 50 proteins, many of which are clinically validated drug targets, have been successfully developed with several in clinical trials for cancer therapy. This article reviews PROTAC-mediated degradation of critical oncoproteins in cancer, particularly those in hematological malignancies. Chemical structures, cellular and in vivo activities, pharmacokinetics, and pharmacodynamics of these PROTACs are summarized. In addition, potential advantages, challenges, and perspectives of PROTAC technology in cancer therapy are discussed.

## Background

Remarkable advances in targeted cancer therapy have been accomplished for the past several decades, and a number of targeted anticancer small-molecule drugs approved for the treatment of various types of cancer. Unlike conventional chemotherapeutics that non-specifically inhibit cell proliferation including that of normal cells and cause undesired toxicities and side effects, a targeted cancer therapeutics suppresses cancer proliferation and progression by interacting with its protein of interest (POI) that cancer cells (but not normal cells) are heavily dependent on. Ideally, it should be more effective without toxicities to normal tissues. In reality, targeted therapeutics still has undesired toxicities and side effects because of selectivity issues: the drug itself is less specific to the POI with off-target activities on other proteins, or the POI is not cancer-specific with physiological functions in normal cells. Another problem for these small molecule-based, protein-interacting agents in the clinic is that cancer can develop resistance. One common mechanism is mutation through which the mutant POI no longer interacts strongly with the drug. Another mechanism of resistance is that cancer can evade or become insensitive to the drug by overexpression of the POI or adapting to an alternative signaling pathway for growth or survival. Given these limitations, strategies have been developed for targeted protein reduction as an alternative approach to cancer therapy.

Targeted protein reduction may be readily accomplished at the transcription level using nucleic acid-based methods [1], including RNA interference (RNAi) [2] and more recently, CRISPR/Cas9-mediated gene knockout technology [3]. However, because nucleic acid-based molecules are unable to passively penetrate into cells and subjected to rapid enzyme-mediated hydrolysis, significant challenges have hampered them from becoming clinically useful drugs, including safe and efficient cell delivery, metabolic stability [4], off-target effects [5], and potential immunogenicity [6]. To date, only 9 nucleic acid-based drugs that inhibit specific protein production in patients have been approved in the USA, and none of which are for cancer therapy [7, 8]. Therefore, small molecules have been explored to reduce a protein in cells, which works at the post-translational level to cause its degradation. In early work, inhibitors of chaperone protein heat shock protein 90 (HSP90) can induce degradation of its client proteins, including many known oncoproteins, in cancer cells. However, although more than 30 of HSP90 inhibitors have been in clinical trials during the past two decades, none have been approved due to their complex pharmacology and poor selectivity of protein degradation [9]. More successfully, selective small-molecule degraders of estrogen receptor (ER) have been discovered and developed, among which fulvestrant [10] has been approved to treat hormone receptor-positive metastatic breast cancer [11, 12]. Mechanistically, these compounds bind to ERα, induce protein conformational changes, and cause its degradation [13]. This strategy is, however, not generally applicable to find degraders targeting other proteins.

Two strategies including hydrophobic tagging (HyT) [14] and proteolysis-targeting chimera (PROTAC) [15] have been developed for degrading a broader range of proteins. An HyT probe is designed and synthesized by covalently attaching a hydrophobic moiety to a ligand of the POI. The binary POI-HyT complex can mimic a partially denatured state for protein degradation [16]. The mostly used hydrophobic moieties include adamantine and BOC_3_-Arg [14, 17, 18]. HyT had limited applications, because BOC_3_-Arg was found to inhibit the mammalian target of rapamycin complex 1 (mTORC1) pathway [19].

PROTAC is the focus of this review because of its well-understood mechanism as well as broad applications with two compounds currently in clinical trials targeting cancer. Several reviews have offered recent advances of this technology [20–23] as well as its application in targeted protein degradation [24–26]. This review is focused on PROTAC-mediated degradation of critical oncoproteins implicated in cancer, particularly in hematological malignancies. Chemical structures, cellular and in vivo activities, pharmacokinetics, and pharmacodynamics of these PROTACs are summarized for cancer therapy. In addition, potential advantages, challenges, and perspectives of PROTAC technology in cancer therapy are discussed.

## What is PROTAC?

**Fig. 1:**
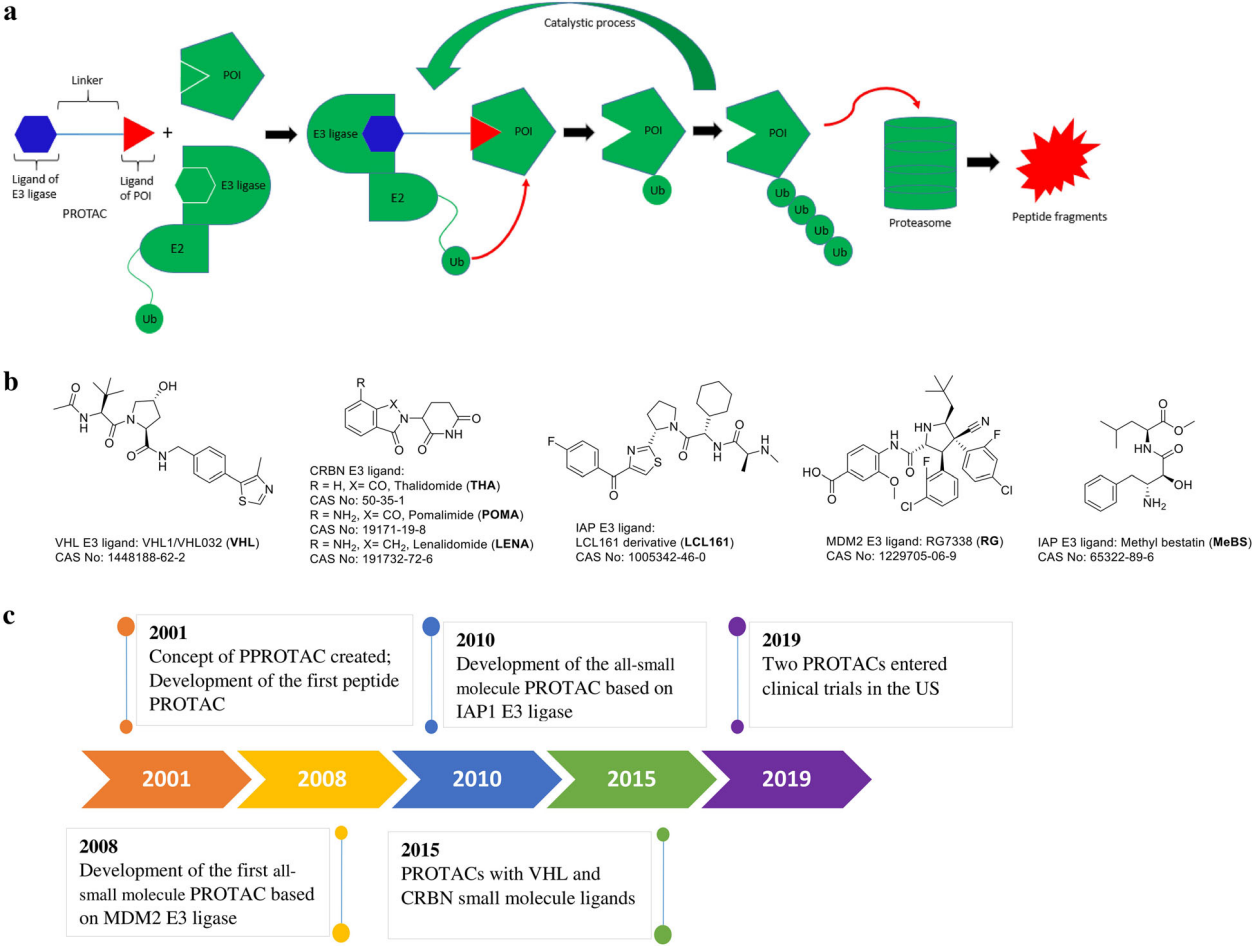
**a** Mechanism of PROTAC-mediated protein degradation. **b** Representative small-molecule ligands of E3s used for PROTAC. **c** Timeline and major milestones for the development of PROTAC

A PROTAC molecule consists of a ligand (mostly small-molecule inhibitor) of the POI and a ligand of an E3 ubiquitin ligase (E3), which are covalently interconnected with a linker of mostly 5-15 carbon or other atoms. Mechanistically as shown in Fig. 1a, upon binding to POI, the PROTAC can recruit E3 for proximity-induced ubiquitination of POI, which is then subjected to degradation by endogenous 26S proteasome. A recent x-ray structure of POI-PROTAC-E3 ternary complex provides strong evidence to support this mechanism [27]. Although there are > 600 E3 ubiquitin ligases, only several with small molecule ligands have been used for designing PROTACs, including Skp1-Cullin-F box complex containing Hrt1 (SCF) [28], Von Hippel-Lindau tumor suppressor (VHL) [29], Cereblon (CRBN) [30], inhibitor of apoptosis proteins (IAPs) [31], and mouse double minute 2 homolog (MDM2) [32]. Representative ligands of these E3s are showed in Fig. 1b. Figure 1c shows the major events and milestones for the development of PROTAC technology.

To evaluate its protein degradation activity, a PROTAC molecule at a range of concentrations is incubated with selected cells expressing the POI for 2 to 24 h. Western blot is typically used to visualize and quantitate the cellular levels of the POI and dose-dependent experiments that give a DC_50_ value (concentration at which the POI is reduced by 50%) for the PROTAC. It is important to choose a wide range of concentrations for activity testing, as PROTACs tend to show Hook effect at a higher concentration: an effective PROTAC typically reduces the POI levels dose-dependently to a certain concentration, while the POI may gradually increase beyond the point, showing a bell-shaped dose-response curve. A representative case can be found in Ref [33]. This is consistent with the mechanism of PROTAC. A high concentration of PROTAC favors the formation of E3-PROTAC and PROTAC-POI binary complexes, while the POI-PROTAC-E3 ternary complex is depleted, showing a decreased degradation activity.

## PROTACs targeting Bromodomain-containing protein 4 (BRD4)

**Fig. 2:**
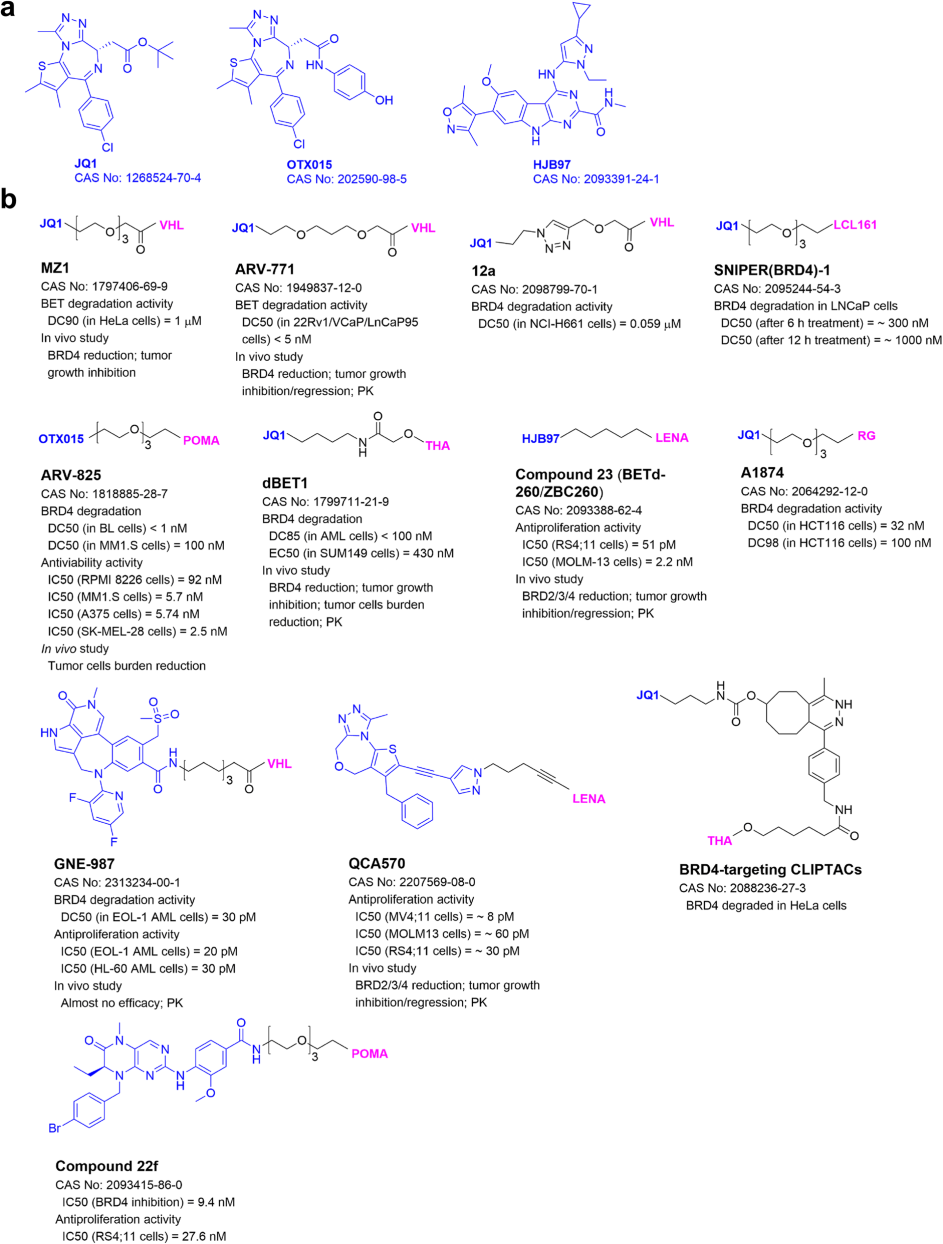
**a** Common ligands of BRD4. **b** Structures and biological activities of PROTACs targeting BRD4. Structures of the E3 ligands are shown in Fig. 1

BRD4, a member of the bromodomain and extra-terminal (BET) family, functions as an essential translation cofactor to regulate gene expression in mammalian cells. It binds to an acetylated lysine residue of histone or a transcription factor and recruits positive transcription elongation factor-b (P-TEFb), which phosphorylates RNA polymerase II for gene transcription [34]. Dysregulated BRD4 and other BET proteins are frequently found in cancer showing aberrant chromatin remodeling and gene expression [35]. Small-molecule inhibitors of BRD4, such as JQ1 and HJB97, have been developed and show antitumor activity [36]. Bradner and collaborators [37] developed the first BRD4-targeting PROATC dBET1 (Fig. 2) with JQ1 and thalidomide as the ligands of BRD4 and CRBN, respectively. It had a sub-micromolar DC_50_ value for BRD4 degradation and significantly inhibited proliferation of MV4;11 leukemia cells in vitro and in a mouse model. As summarized in Fig. 2, more BRD4-targeting PROTACs with antitumor activities have been developed.

### VHL-based PROTACs

VHL1 (Fig. 1b) is commonly used as the ligand of VHL-based PROTACs. Pan-BET inhibitor JQ1 containing MZ1 (Fig. 2), developed by the Ciulli group, showed potent protein degradation activity as well as selectivity for BRD4 over BRD2/3 [38]. It also strongly inhibited tumor growth as well as induced BRD4 degradation in a mouse model of JQ1-resistant triple negative breast cancer [39]. Macrocyclic PROTAC-1, a derivative of MZ1, showed strong and selective BRD4 degradation and potent anti-proliferation activity in several leukemia cells [40]. In addition, x-ray structure of the first PROTAC ternary complex BRD4-MZ1-VHL was determined [27], supporting the mechanism of action for PROTAC.

JQ1-containing ARV-771 (Fig. 2), developed by Arvinas LLC and collaborators, exhibited potent and selective BRD4 degradation and more potent anti-proliferation activity than its parent inhibitor [41]. In a mouse model of castration-resistant prostate cancer, ARV-771 induced significant BRD4 degradation and showed potent antitumor activity with low toxicity. As compared to JQ1, ARV-771 showed more pronounced antitumor activity in a mouse model of acute myeloid leukemia and prolonged animal survivals [42]. PhotoPROTAC-1 [43] was derived from ARV-771 with a light-switchable linker, which upon irradiation, can undergo a cis- to trans-isomerization. While the cis-isomer is inactive, the trans-isomer with the desired stereochemistry is active in BRD4 degradation in Burkitt lymphoma Ramos cells.

GNE-987 (Fig. 2), containing a potent tetracyclic BRD4 inhibitor, exhibited extremely high activities in degradation of BRD4 and growth inhibition of acute myeloid leukemia EOL-1 cells in low pM levels [44]. Due to its poor pharmacokinetics (PK), GNE-987 was attached to an antibody of CLL1 (C-type lectin-like molecule-1) to form the conjugate CCL1-5 with good PK properties. CCL1-5 showed significant antitumor activities without severe toxicity in mouse models of acute myeloid leukemia. Using a click chemistry, JQ1-containing PROTAC 12a (Fig. 2) was generated and potently degraded BRD4 in lung cancer NCI-H661 cells [45].

### CRBN-based PROTACs

Thalidomide and its analogs (Fig. 1b) are common ligands of E3 ligase CRBN. ARV-825 (Fig. 2), consisting of pomalidomide and BRD4 inhibitor OTX015, induced degradation of BRD4 and inhibited proliferation of Burkitt lymphoma cells at sub-nanomolar levels [33]. It also showed potent activities to degrade BRD4 and inhibited cell proliferation in patient-derived secondary acute myeloid leukemia [42], triple negative breast cancer, ovarian cancer [39], and multiple myeloma cells [46]. In a multiple myeloma mouse model, ARV-825 exhibited in vivo antitumor activity without overt toxicities. To improve solubility, ARV-825 was loaded into a self-nanoemulsifying preconcentrate (ARV-SNEP) [47], which had low nanomolar EC_50_s against proliferation of vemurafenib-resistant melanoma cells.

dBET6 [48], a linker optimized derivative of dBET1, showed more activities in BRD4 degradation and tumor growth inhibition in cells and mouse models of acute lymphoblastic leukemia. PROTAC-I-3 was derived from dBET1 with a light-switchable linker [49]. Upon irradiation, it robustly decreased BRD4 in leukemia RS4;11 cells and showed a potent anti-proliferation activity. Pc-PROTAC1 is a photo-caged PROTAC, which is stable in the dark, but can release dBET1 upon irradiation and induce BRD4 degradation in cells and in vivo [50]. Another photo-caged PROTAC4 induced BRD4 degradation in HEK293T cells and reduced the viability of prostate carcinoma 22Rv1 cells upon irradiation [51].

Compound 23 (Fig. 2) [52] with lenalidomide to recruit CRBN and HJB97 to bind to BET proteins can induce degradation of BRD2/3/4 with DC_50_ values of 30–100 pM and showed highly potent antitumor activities in cell and mouse models of several leukemias. BETd246, an analog of compound 23, showed potent BRD2/3/4 degradation and anti-proliferative activities in triple negative breast cancer cells [53]. BETd246 also effectively inhibited tumor growth in a mouse model of patient-derived, treatment-resistant breast cancer without overt toxicities to the animals. However, BETd246 did not have antitumor activity in mouse models of breast cancer MDA-MB-231 and -468, while compound 23 with more exposure in the tumor tissues showed in vivo antitumor activity. QCA570 (Fig. 2) containing a potent inhibitor QCA276 induced degradation of BET proteins and inhibited cell growth at pM levels in several leukemia cells [54]. In a xenograft mouse model of leukemia, administration of QCA570 resulted in tumor regression without severe toxicity. In addition, compound 22f with pomalidomide and BRD4 inhibitor BI2536 exhibited potent BRD4 inhibitory activity as well as BRD4 degradation activity [55]. It also showed potent activity against growth of RS4;11 leukemia cells.

In-cell click-formed PROTAC (CLIPTAC)-targeting BRD4 (Fig. 2) was developed using intracellular click reaction between the tetrazine-tagged thalidomide and the trans-cyclo-octene-tagged JQ1 [56]. Treatment of HeLa cells with the two agents led to a complete degradation of BRD4.

### IAP-based PROTACs

SNIPER(BRD4)-1 (Fig. 2) with LCL161 and JQ1 to recruit IAP E3 ligase and BRD4-induced BRD4 degradation at 3 nM in prostate cancer LNCaP cells [57].

### MDM2-based PROTACs

A1874 (Fig. 2) with RG7338 as the MDM2 ligand significantly induced degradation of BRD4. It increased the p53 level in colon cancer HCT116 cells, due to the retained activity of RG7338 against MDM2 [58]. A1874 potently inhibited proliferation of p53-wild-type cancer cells, presumably due to dual inhibition of BRD4 and MDM2.

## PROTACs targeting Bruton’s tyrosine kinase (BTK)

**Fig. 3:**
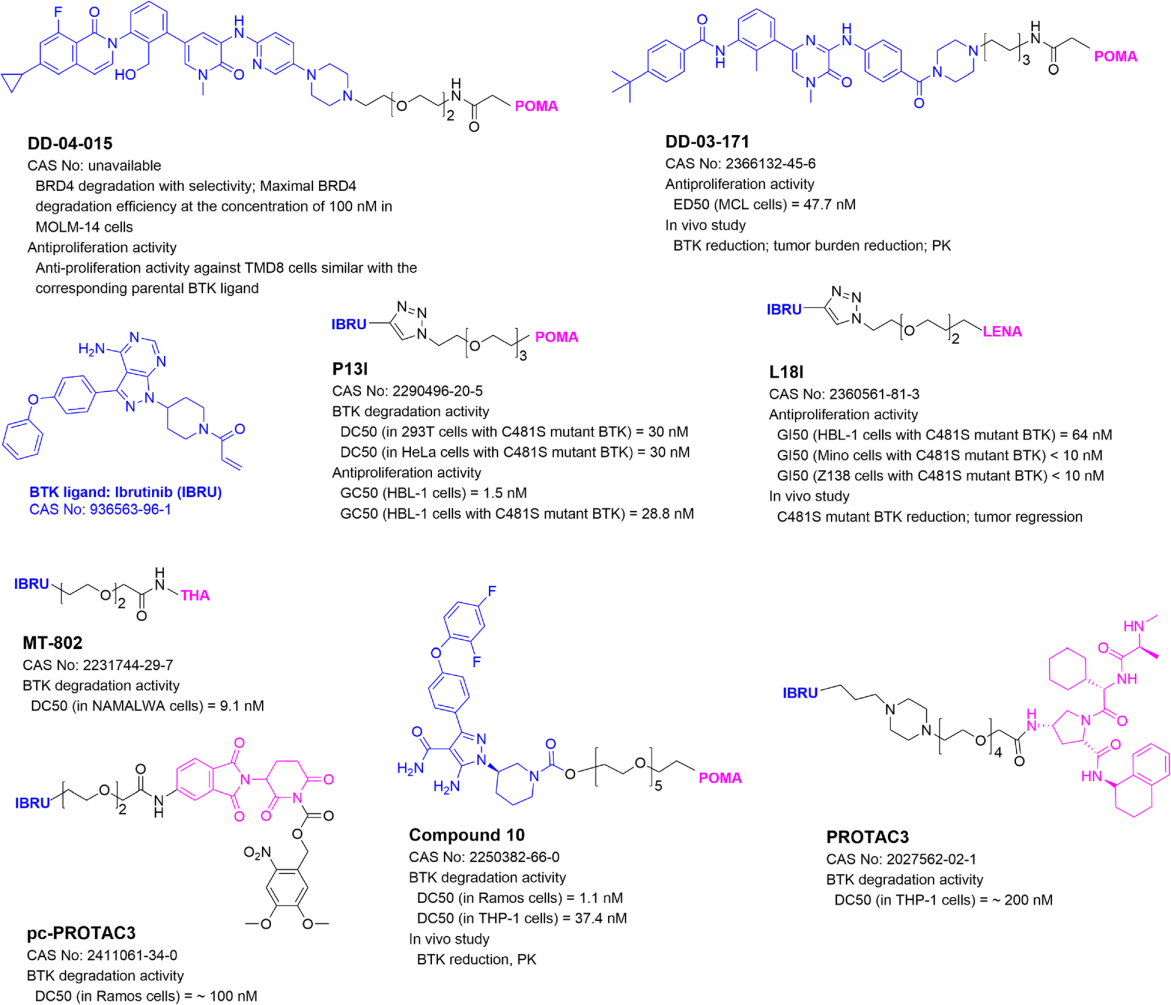
Structures and biological activities of PROTACs targeting BTK

Predominantly expressed in hematopoietic cells [59], BTK is a non-receptor tyrosine kinase playing essential roles in B-cell development, differentiation, and signaling. BTK is closely associated with survival and proliferation of B-cell neoplasms via B-cell receptor (BCR) signaling [60]. Antigenic stimulation of BCR triggers translocation of BTK from cytosol to the plasma membrane, where BTK is phosphorylated and activated by the Src family kinases. BTK drives multiple pro-survival and proliferative pathways, including AKT, ERK, and NF-кB pathways, to enhance survival and promote proliferation. Gray and collaborators [61] developed the first BTK-targeting PROTAC D-04-015 (Fig. 3), which consists of pomalidomide and the BTK ligand RN486. It efficiently and selectively degraded BTK and inhibited proliferation of B-cell lymphoma TMD8 cells with a comparable activity to its parent inhibitor.

### CRBN-based PROTACs

DD-03-171 (Fig. 3) with pomalidomide and BTK inhibitor CGI1746 not only caused selective degradation of wild-type BTK [62], but it is also active in degradation of ibrutinib-resistant, C481S mutant BTK. It exhibited comparable anti-proliferative activities in sensitive or resistant B-cell lymphoma cells. In addition, DD-03-171 significantly inhibited tumor growth and prolonged animal survival in mouse models of mantle cell lymphoma. MT-802 was developed based on thalidomide and Ibrutinib. It exhibited activities in degradation of wild-type and mutant BTK and inhibited cell proliferation in Burkitt lymphoma and chronic lymphocytic leukemia cells [63]. A photo-caged PROTAC analog pc-PROTAC3 exerted similar activities upon irradiation [50].

P13I (Fig. 3) based on ibrutinib and pomalidomide showed low nanomolar DC_50_ values in selective BTK degradation and cell growth inhibition of non-Hodgkin’s lymphoma cells [64]. Moreover, it inhibited growth of diffuse large B-cell lymphoma cells having wild-type and C481S mutant BTK with a similar potency. L18I, a more soluble PROTAC with lenalidomide, was able to induce degradation of BTK with multiple mutations in HeLa cells with an average DC_50_ value of 30 nM, and inhibited growth of lymphoma cells harboring C481S mutant BTK in vitro and in vivo [65].

Compound 10 (Fig. 3) having pomalidomide and a phenyl-pyrazole-based BTK inhibitor exhibited potent and selective activity in degradation of BTK in Burkitt lymphoma Ramos and leukemia THP-1 cells [66]. Interestingly, in vivo studies in rats revealed that compound 10 induced efficient BTK degradation in spleen but not in lungs, despite similar drug delivery to both organs. This shows PROTAC-mediated protein degradation can be tissue-selective, which is of significance in the perspective of cancer therapy and deserves more in-depth investigation.

### IAP-based PROTACs

Tinworth and coworkers [67] studied the effects of covalent and non-covalent binding of PROTACs to BTK with covalent inhibitor ibrutinib as well as a reversible analog. PROTAC3 (Fig. 3), with a reversible BTK binding ligand, potently degraded BTK with a DC_50_ of 200 nM, while the covalent PROTAC failed to induce BTK degradation.

## PROTACs targeting BCR-ABL

**Fig. 4:**
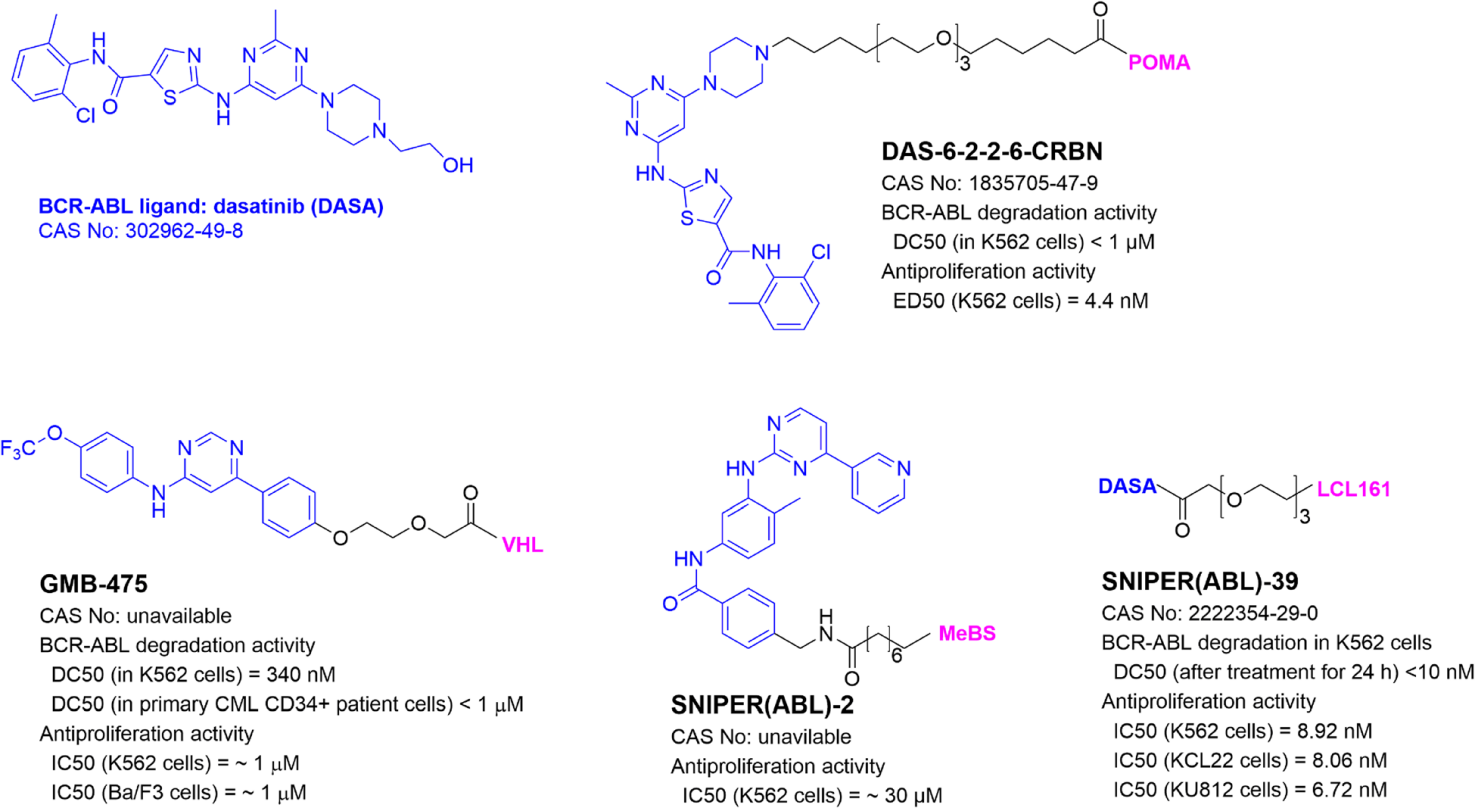
Structures and biological activities of PROTACs targeting BCR-ABL

Fusion oncoprotein BCR-ABL, generated by chromosome translocation t(9;22)(q34;q11), is a constitutively active tyrosine kinase [68], whose activity leads to oncogenesis of chronical myeloid leukemia (CML). Small molecule inhibitors of BCR-ABL, such as imatinib and dasatinib, are successfully used to treat the malignancy. However, BCR-ABL mutations can cause drug resistance and treatment failure [69]. PROTAC has been developed to overcome the drug resistance or as an alternative treatment. The Crews group [70] synthesized the first BCR-ABL-targeting PROTAC DAS-6-2-2-6-CRBN (Fig. 4) containing pomalidomide and dasatinib. It exhibited a high potency in BCR-ABL degradation and growth inhibition in CML K562 cells.

GMB-475 (Fig. 4) containing BCR-ABL inhibitor GNF5 can induce degradation of BCR-ABL in CML K562 cells [71]. As compared to imatinib, anti-proliferation activity of GMB-475 was less affected in cells with imatinib-resistant BCR-ABL mutants. Furthermore, GMB-475-induced degradation of BCR-ABL at sub-micromolar levels in primary CML patient cells inhibited their proliferation, while it was not toxic to normal CD34+ cells from healthy donors. SNIPER(ABL)-2 (Fig. 4), containing imatinib and MeBS to recruit E3 ligase IAP, showed strong BCR-ABL degradation ability and potently inhibited growth of CML K562 cells [72]. Its derivative SNIPER(ABL)-39 with dasatinib and LCL161 for IAP recruitment was found to have more potent BCR-ABL degradation activity and showed high activities against proliferation of several CML cells with EC_50_ values of ~ 8 nM [57, 73].

## PROTACs targeting MCL1

**Fig. 5:**
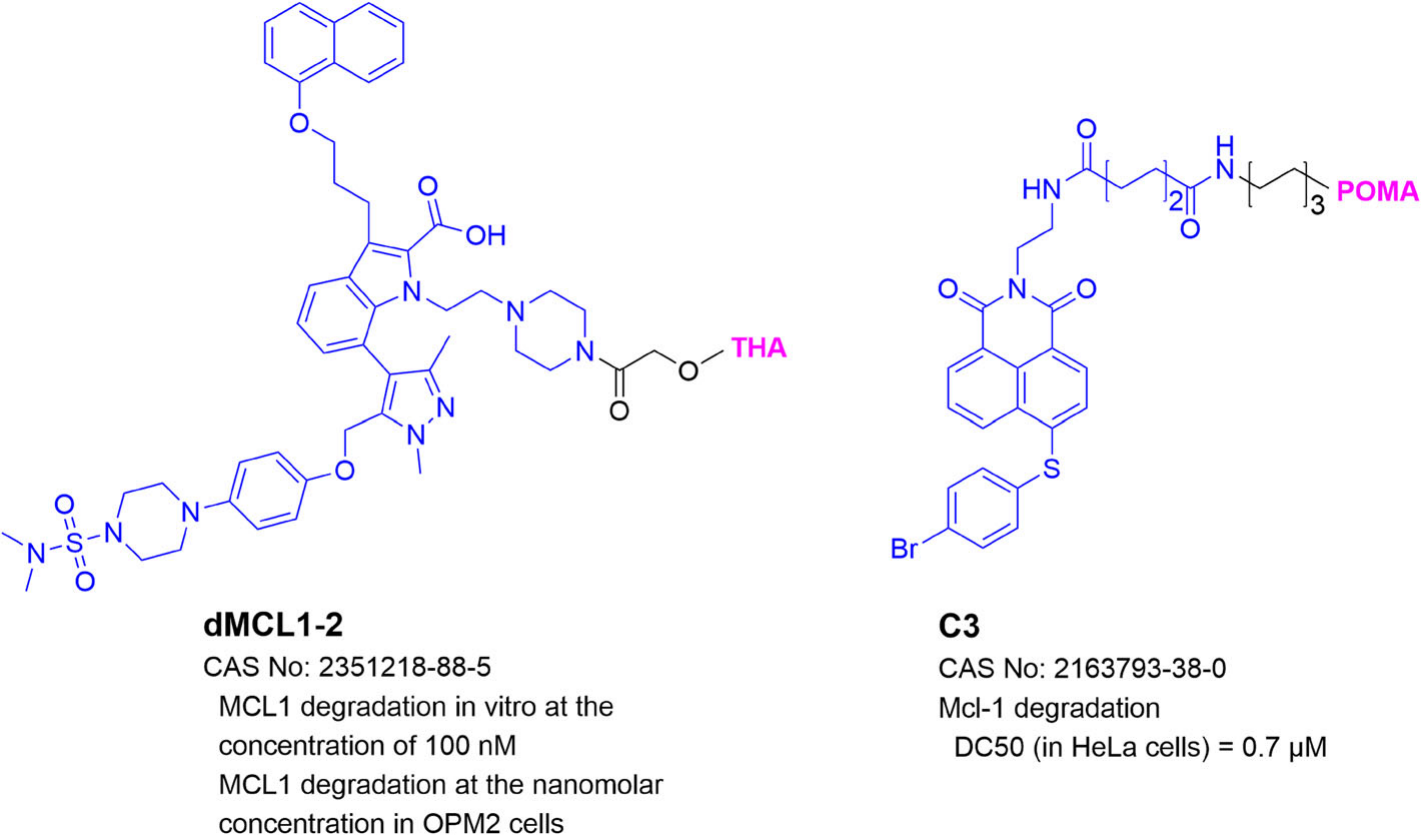
Structures and biological activities of PROTACs targeting MCL1

MCL1 is a pro-survival protein in the B-cell lymphoma 2 (BCL2) family [74]. It contains three BH domains forming a hydrophobic groove that binds to BH3-containing proteins, including other pro-apoptotic Bcl-2 family members Noxa, Bad, Bim, Bak, and Bcl-2-associated protein X (Bax). The protein-protein interactions of MCL1 with these proteins repress conformational activation of Bak/Bax and inhibit the release of cytochrome c from mitochondria into the cytoplasm, which activates the caspase cascade and leads to apoptosis of the cell [75]. MCL1 overexpression has been identified as a vital survival factor in lymphoma, leukemia, and multiple myeloma [75]. Therefore, degradation of MCL1 represents a novel therapeutic approach for these cancers. The first MCL1-targeting PROTAC dMCL1-2 (Fig. 5) contains thalidomide and an MCL1 inhibitor A-1210477, which can successfully degrade MCL1 at nM concentrations in multiple myeloma OPM2 cells [76]. Compound C3 (Fig. 5) with pomalidomide and an MCL1 inhibitor Nap-1 induced MCL1 degradation with a DC_50_ of 0.7 μM [77]. C3 exhibited more potent anti-proliferative activity than MCL1 inhibitors Nap-1 and A-1210477.

## PROTACs targeting FMS-like tyrosine kinase 3 (FLT-3)

FLT-3 is a receptor tyrosine kinase and primarily expressed in hematopoietic progenitor cells and dendritic cells [78]. FLT-3 plays key roles in regulating early hematopoiesis. Mutations of FLT-3 are frequently found in acute myeloid leukemia (AML) [79], which cause constitutive activation of FLT-3 and induce activation of multiple downstream signaling pathways, including signal transducers of activation and transcription (STATs), RAS, mitogen-activated protein kinases (MAPKs), and phosphatidyl inositol-3 kinase (PI3K)/AKT pathways. These events suppress differentiation and apoptosis [80, 81]. FLT-3 bearing an internal tandem duplication (ITD) mutation has been validated to be a driving factor for AML [79]. Several FLT-3 inhibitors have been developed and in clinical trials for AML therapy showing, however, limited clinical benefits. One of the reasons could be that these FLT-3 inhibitors seem to increase or stabilize the protein [61, 82]. Therefore, targeted FLT-3 degradation could be an effective therapy.

**Fig. 6:**
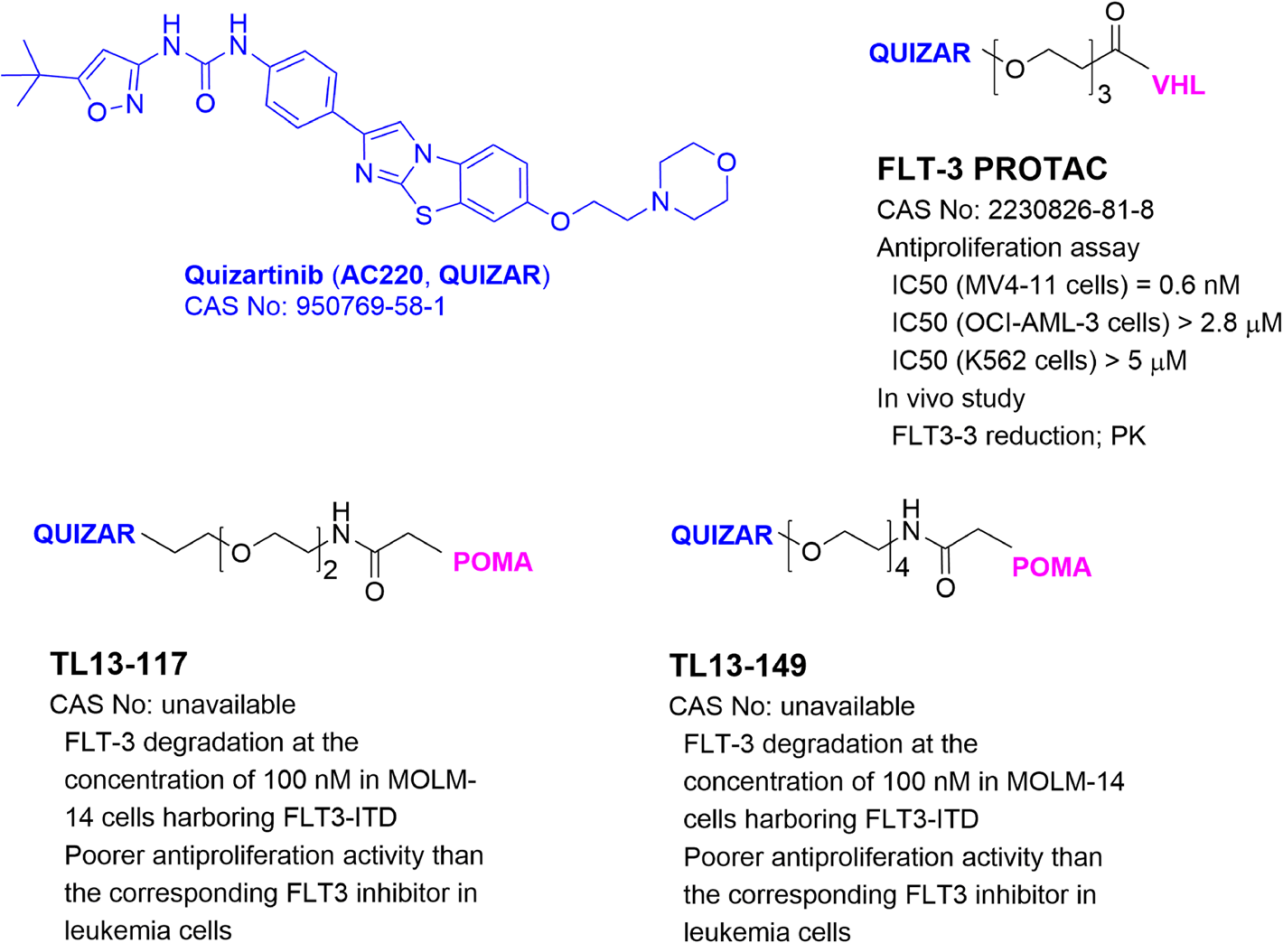
Structures and biological activities of PROTACs targeting FLT-3

Gray and collaborators [61] developed the first FLT-3-targeting PROTACs TL13-117 and TL13-149 (Fig. 6) using pomalidomide and FLT-3 inhibitor quizartinib. These probes reduced cellular FLT-3 levels in leukemia MOLM-14 cells harboring FLT3 ITD mutation. However, they showed less activity against leukemia cell proliferation as compared to their parent inhibitor. Another FLT-3 PROTAC (Fig. 6) with VHL1 and quizartinib is more potent. It induced degradation of FLT-3 ITD at low nM concentrations [83] and inhibited proliferation of the leukemia cells at < 1 nM, while it showed less activities in cells with D835Y or F691L-mutated FLT-3. Furthermore, FLT-3 PROTAC had good PK properties and can decrease FLT-3 protein in tumor tissues in a mouse model of MV4-11 leukemia.

## PROTACs targeting STAT3

Transcription factor STAT3 mediates signal transduction from a cell surface receptor to the nucleus [84]. Upon receiving extra- or intra-cellular stimuli, STAT3 in the cytosol is phosphorylated and dimerized to become activated STAT3, which is then translocated into the nucleus, binds to its target DNA sequences in the gene promoters, and starts gene transcription [85]. STAT3 plays important roles in regulating cell differentiation, development, proliferation, and apoptosis [86, 87]. In particular, it mainly regulates expression of diverse genes involved in cancer cell survival, proliferation, invasion, and drug resistance. STAT3 is therefore an attractive therapeutic target for the treatment of cancer and other diseases [85, 86].

**Fig. 7:**
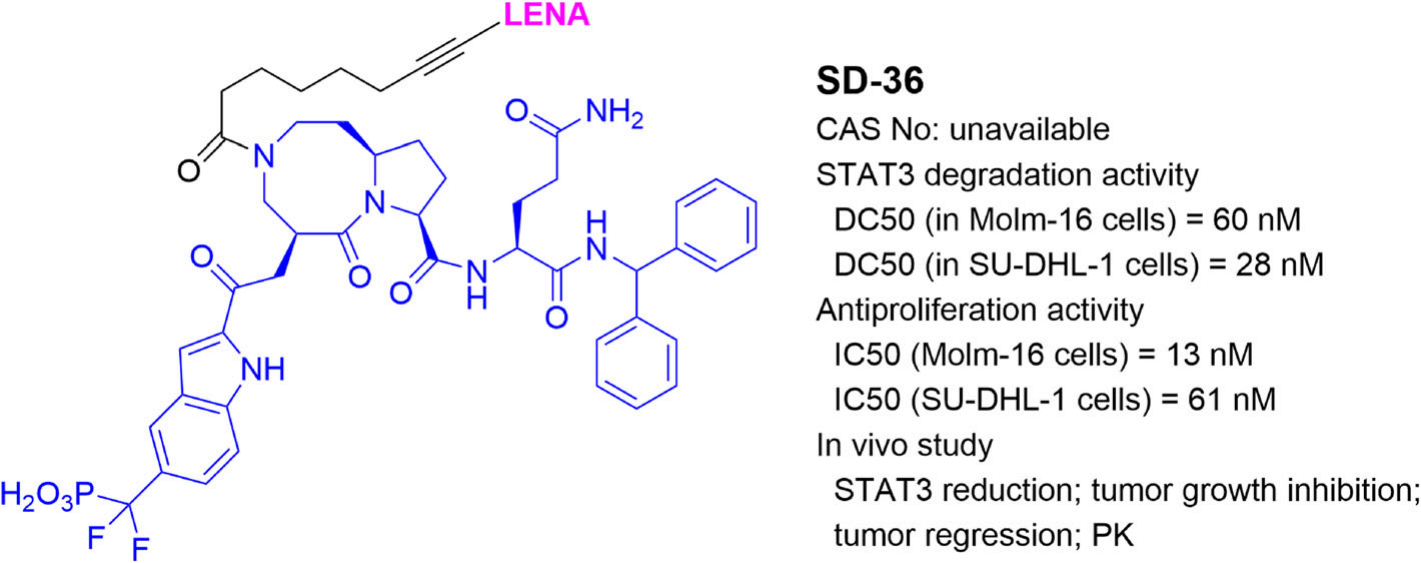
Structure and biological activities of PROTAC targeting STAT3

Wang and coworkers [88, 89] developed a highly potent STAT3 inhibitor SI-109 and used it to develop a STAT3-targeting PROTAC SD-36 (Fig. 7). At low nM concentrations, SD-36 efficiently reduced STAT3 in a number of leukemia and lymphoma cells. It showed high selectivity for STAT3 over other STAT family members. SD-36 also exhibited potent antitumor activities in these cancer cells and in a mouse model of Molm-16 leukemia without overt toxicities.

## PROTACs targeting Brg/Brahma-associated factors (BAF complex)

BAF complex, as a member of the ATP-dependent chromatin remodeling complex family, plays important roles in the regulation of gene expression and differentiation. This protein complex contains up to 15 subunit proteins, many of which can be replaced by their paralogs. This leads to hundreds of possible combinations of assemblies in mammalian cells [90]. Compositions of a BAF complex depend on a distinct development stage or different tissue type [90]. In leukemia, the BAF complex is assembled around the Brg ATPase, which is necessary for leukemia progression. Brg ATPase inactivation or knockdown showed therapeutic benefits in AML [91].

**Fig. 8:**
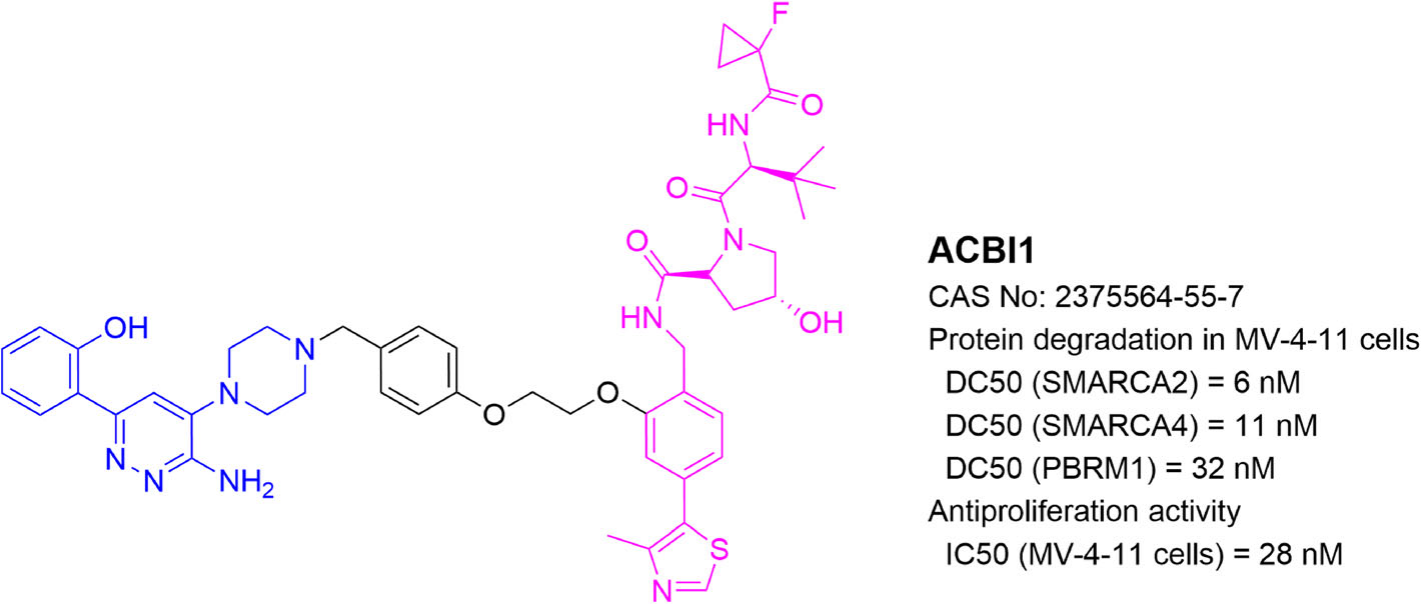
Structure and biological activities of PROTAC targeting BAF

Ciulli  and collaborators [92] reported a BAF-targeting PROTAC ACBI1 (Fig. 8), using VHL1 and a small molecule ligand of SMARCA, a subunit protein of the BAF complex. ACBI1 potently induced a complete degradation of SMARCA2/4 in leukemia MV-4;11 cells as well as in SMARCA4-deficient human non-small cell lung carcinoma NCI-H1568 cells. Moreover, ACBI1 potently inhibited proliferation of a panel of cancer cells such as leukemia MV-4;11 and melanoma SK-MEL-5.

## PROTACs in clinical trials

Recently, two PROTAC probes, ARV-110 and ARV-471 (with undisclosed structures) developed by Arvinas LLC, have been in phase І clinical trials (NCT03888612 and NCT04072952 in clinicaltrials.gov) for prostate and breast cancer, respectively [93, 94]. ARV-110 targets androgen receptor (AR). It was found to degrade the wild-type protein as well as multiple clinically relevant AR mutants with DC_50_ values of ~ 1 nM. In VCaP cells, ARV-110 strongly inhibited cell proliferation as well as induced robust apoptosis. In a castrated mouse model of VCaP prostate cancer, treatment with ARV-110 at 1 mg/kg, p.o., once a day for 3 days, induced degradation of > 90% AR at 16 h post-treatment. Furthermore, ARV-110 also showed high activities in castrated mice bearing LNCaP and enzalutamide-resistant VCaP prostate cancer xenografts.

ARV-471-targeting ERα was found to efficiently degrade the wild-type and clinically relevant ERα mutants (Y537S and D538G) with DC_50_ values of ~ 2 nM in multiple ER-positive breast cancer cell lines. In mouse models of MCF7 breast cancer, treatment with ARV-471 in a dose as low as 3 mg/kg, p.o., daily, led to tumor regression together with > 90% of ER reduction in the tumor tissues. Combination therapy of ARV-471 with a CDK4/6 inhibitor showed more pronounced antitumor activity. Moreover, in PDX models of hormone-independent breast cancer with ERα mutations, treatment with ARV-471 in a dose of 10 mg/kg completely inhibited tumor growth accompanied with significantly reduced mutant ER levels.

## Perspectives and conclusions

PROTAC, first described by Crews and coworkers in 2001 [15], has been successfully developed to be a useful technology for targeted degradation of ~ 50 proteins, most of which are clinically validated drug targets. It complements nucleic acid-based gene knockdown/out for targeted protein reduction and could recapitulate the biological activities of pharmacological protein inhibition. The PROTAC technology offers a number of potential advantages, while it also faces significant challenges in the perspectives of cancer therapy.

First, despite their relatively large molecular weights, PROTACs are more drug-like, which is in contrast to RNA/DNA-based protein reduction agents. By choosing drug-like ligands of POI and E3 followed by medicinal chemistry optimization, PROTACs can have good ADME (absorption, distribution, metabolism, and elimination) properties, which are required to become a clinically useful drug. Second, PROTAC may eliminate the POI sub-stoichiometrically, because it can be reused after one round of protein degradation (Fig. 1). It is therefore possible that the DC_50_ of a PROTAC can be significantly lower than its binding affinity (or inhibitory IC_50_) to the POI. For example, as low as 10 pM of a PROTAC can efficiently induced BRD4 degradation [54]. This feature provides a potentially huge advantage over pharmacological protein inhibition. Third, since PROTAC could only require a transient binding to the POI, it provides an opportunity to overcome mutation-directed drug resistance. For example, ibrutinib-containing PROTAC MT-802 induced degradation of C481S mutant BTK (which is resistant to ibrutinib) as effectively as the wild-type protein, and potently inhibited proliferation of the ibrutinib-resistant leukemia cells [63]. Fourth, PROTAC only requires a ligand that binds to the POI, which may not necessarily affect POI’s function. Therefore, PROTAC can possibly target any proteins, including those considered undruggable. Moreover, PROTAC-induced degradation also depends on the lysine residues on the POI surface, which represent additional selectivity requirements. This might lead to a higher selectivity and has been successfully used to develop selective PROTACs targeting an isoform of a protein family, such as CDK9 [95], BRD4 [38], and HDAC6 [96], starting from a pan-inhibitor of the protein family.

On the other hand, there are significant challenges for PROTAC to be a successful drug development approach. First, unlike nucleic acid-based methods which are routinely performed using commercially available agents to knockdown or knockout a POI, the major challenge for PROTAC technology is the uncertainty, difficulty, and high costs, even when there are available ligands/inhibitors of the POI. Enormous amount of medicinal chemistry, biochemistry, and cell biology studies is needed to optimize the site of linkage, the linker, and the E3 ligand of the PROTAC. Unfortunately, these efforts may not guarantee a success. Only a limited number (~ 50) of POI-PROTACs have been reported to date. Second, biological activities of a PROTAC, which reduces the POI, may be different from those caused by pharmacological inhibition of the POI. Therefore, it is unreliable to predict the biological or clinical outcomes of a PROTAC based on the POI inhibitor it contains. Third, because of their relatively large molecular weights (mostly > 800), ADME properties of PROTACs could be different from small-molecule drugs (typically < 500). Fourth, PROTAC’s activity is dependent on its associated E3, whose expression may vary in different cell types, tissues, or species [70]. In a recent report [66], a CRBN-based PROTAC 10 (Fig. 3) showed a drastically different BTK degradation efficacy in rat spleen and lung, even though the distribution and uptake of this PROTAC were similar in these tissues. Fourth, PROTAC technology has possible off-target effects related to its E3 ligand moiety. However, given the well-studied pharmacology and toxicology of these ligands (e.g., thalidomide and its analogs), these off-target effects may be predicted with no significant toxicity. For example, CRBN ligand thalidomide and its analogs are used in the clinic to treat multiple myeloma. These drugs are generally inactive to irrelevant tumor and normal cells, although their binding to CRBN may show certain biological effects [97]. MDM2-based PROTACs could block the interactions between MDM2 and p53 and show related biological activities [98]. Finally, cancer can also develop resistance to a PROTAC with a different mechanism [99].

In summary, PROTACs targeting ~ 50 proteins have been successfully developed to date, among which two compounds are currently in clinical trials to treat therapy-resistant prostate and breast cancer. No clinical outcomes have been disclosed. Given these relatively small numbers of POIs and clinical candidates, it remains to be seen whether these PROTACs can become clinically useful anticancer drugs. However, the PROTAC technology is far from well explored and developed. It has a great potential in the perspective of cancer therapy. There are > 600 E3 ubiquitin ligases in human, and many of them may be used for designing a PROTAC [100]. Success in this aspect, together with more understanding of the functions and tissue-specific expression of these E3s, could greatly broaden the feasibility, utility, and selectivity of the PROTAC technology [70]. Moreover, development of peptidomimetic-based PROTACs could be a useful alternative [101].

## Data Availability

Not applicable.

## Abbreviations

PROTAC: Proteolysis-targeting chimera
POI: Protein of interest
E3: E3 ubiquitin ligase
RNAi: RNA interference
HSP90: Heat shock protein 90
ER: Estrogen receptor
HyT: Hydrophobic tagging
mTORC1: Mammalian target of rapamycin complex 1
SCF: Skp1-Cullin-F box complex containing Hrt1
VHL: Von Hippel-Lindau tumor suppressor
CRBN: Cereblon
IAPs: Inhibitor of apoptosis proteins
MDM2: Mouse double minute 2 homolog
BRD4: Bromodomain-containing protein 4
BET: Bromodomain and extra-terminal family
P-TEFb: Positive transcription elongation factor-b
CLL1: C-type lectin-like molecule-1
BL: Burkitt’s lymphoma
MCL1: Myeloid cell leukemia 1
CLIPTACs: Click-formed proteolysis targeting chimeras
BTK: Bruton’s tyrosine kinase
BCR: B-cell receptor
CLL: Chronic lymphocytic leukemia
Bak: Bcl-2 homologous antagonist killer
Bax: Bcl-2-associated protein X
FLT-3: FMS-Like tyrosine kinase 3
STATs: Signal transducers of activation and transcription
MAPKs: Mitogen-activated protein kinases
PI3K: Phosphatidyl inositol-3 kinase
ITD: Internal tandem duplication
BAF complex: Brg/Brahma-associated factors
PK: Pharmacokinetic
PD: Pharmacodynamics
AR: Androgen receptor
